# Editorial: Biomarkers and therapeutic strategies in acute lymphoblastic leukemia

**DOI:** 10.3389/fcell.2023.1211569

**Published:** 2023-05-17

**Authors:** Ki-Young Lee, Maristella Maggi, Claudia Scotti

**Affiliations:** ^1^ Department of Cell Biology and Anatomy, Arnie Charbonneau Cancer and Alberta Children’s Hospital Research Institutes, University of Calgary, Calgary, AB, Canada; ^2^ Unit of Immunology and General Pathology, Department of Molecular Medicine, University of Pavia, Pavia, Italy

**Keywords:** acute lymphoblastic leukemia, biomarkers, chemotherapy, blood-related disorders, leukemia

Acute lymphoblastic leukemia (aLL) is a malignancy characterized by an expeditious increase in immature B- (in ∼85% of cases) and T-(in ∼15% of cases) lymphocytes in the blood and bone marrow. It is the most prevalent cancer in children and the primary cause of death from pediatric cancer (Hunger and Mullighan, 2015). Chemotherapy continues to be the main treatment for aLL (Lee et al., 2019). There is considerable evidence for superior outcome from multiple rounds of highly intensive chemotherapy (Pui and Evans, 2006). However, the risk of acquiring resistance and toxicity from chemotherapy could be fatal. Although novel therapies such as monoclonal antibodies have been developed, their effectiveness is greatly enhanced when used in combination with chemotherapy. Thus, there is continued interest in chemotherapy. However, some of these combinations are effective in certain patients but have no clinical benefit to others, and their non-specific effects make them intolerable to many. Thus, unnecessary toxicity- and resistance-induced patient suffering or mortality occurs frequently, and relapsed and refractory aLL continue to be a major concern. A growing number of resistance biomarkers for aLL drugs have been identified (Kang et al., 2017; Lee et al., 2019), increasing the current understanding of the molecular mechanisms by which chemotherapy resistance develops. On the other hand, the extensive genetic heterogeneity in B- and T-acute lymphoblastic leukemia precursor cells indicates a range of biomarkers that promote disease development and recurrence.

The objective of this Research Topic is to bring forth recent advances in biomarkers and potential therapeutic strategies in aLL as well as their implications in managing the disease. The ultimate goal is to foster exploitation of these discoveries and provide insight into the development of more innovative and effective therapeutic approaches for aLL.

## 1 Novel biomarkers and therapeutics

Utilizing novel innovative tools such as high throughput small molecular drug screening and gene expression analyses/datasets, a number of new biomarkers and potential therapeutics have been identified. For example, a large-scale screening of small molecule drugs for aLL performed by (Nevado et al.) led to the identification of synthetic oleanane triterpenoids (OTs) as sensitizers of T-aLL cells to doxorubicin, a common component of aLL chemotherapy regimens. OTs attenuate the *MYB* transcription factor and its downstream targets, and downregulate the expression of DNA repair genes. The authors proposed that OTs may be utilized in the treatment of aggressive T-aLL with poor prognosis.

Through gene expression analyses of 207 children with high-risk pre-B-aLL, (Ogana et al.) found that upregulation of Artemis, an endonuclease in V(D)J recombination and non-homologous end joining (NHEJ) of DNA double-strand break (DSB) repair, is correlated with poor prognosis. Artemis inhibition sensitizes RAG-expressing T- and B-aLL cells by causing DSBs. Through a large Artemis targeted drug screen, the authors identified four compounds (8,27,171, 8,27,032, 8,26,941, and 8,25,226) that markedly reduce proliferation of B-aLL cells, while sparing normal B Cells. This provides a basis for the authors to suggest that Artemis inhibition as a therapeutic strategy for B-aLL.

Alsuwaidi et al. analyzed datasets to identify genetic biomarkers for early detection, risk stratification, and prognosis in childhood B-aLL. The authors found that *ADAM6* is a novel biomarker for B-aLL development and progression.

Torres-López et al. demonstrate that T-aLL cells express G-protein–coupled estrogen receptor (GPER), which is stimulated by the non-steroidal agonist, G-1. The authors show that G-1 kills T-aLL cells by causing a premature rise in intracellular Ca^2+^ and arresting cells in G_2_/M, resulting in apoptosis. They suggest that G-1 is a promising T-aLL therapeutic drug.

Mukherjee et al. tackled early T precursor-aLL (ETP-aLL), which still exhibits poor clinical outcome. Gene expression of ETP-aLL blasts was compared to non-ETP-aLL blasts and nine genes (KIT, HGF, NT5E, PROM1, CD33, ANPEP, CDH2, IL1B, and CXCL2) were validated as possible diagnostic ETP-aLL markers using another gene expression dataset (GSE78132) with 17 ETP-aLL and 27 non-ETP-aLL samples. B-lineage-skewed markers were also identified, with distinct functionality and possible druggability in ETP-aLL. These data could help identifying the dominant lineage specification programmes in the ETP-aLL blasts on a personalized level, thus providing new drug targets.

Zhang et al. studied NRAS mutations, which affect relapse susceptibility in the ∼15% of aLL cases with negative outcomes. About one-third of the NRAS mutations significantly transformed Ba/F3 cells as measured by IL3-independent growth and, through a high-throughput drug screening method, the authors uncovered that leukemogenic-NRAS mutations might respond to MEK, autophagy, Akt, EGFR signaling, Polo−like kinase, Src signaling, and TGF-β receptor inhibition depending on the mutation profile.

## 2 Novel tools for assessing prognosis

Lu et al. studied the role of immune genes in the prognosis and microenvironment of acute myeloid leukaemia (AML) by single-cell analysis. Comparison of the gene expression between high and low immune cell infiltration clusters identified nine prognostic immune genes. The authors validated a constructed model that can serve as a new method to assess the prognosis of AML patients.

Pan et al. built a novel prognostic model to improve risk stratification in chronic lymphoid leukaemia patients based on ferroptosis, a lipid peroxidation–induced cell death. Using the ferroptosis-related prognostic score (FPS) model, which was based on nine ferroptosis-related genes (FRGs: AKR1C3, BECN1, CAV1, CDKN2A, CXCL2, JDP2, SIRT1, SLC1A5, and SP1), they showed that patients with high FPS correlated with worse overall and treatment free survival.

## 3 Allogeneic hematopoietic stem cell transplantation (allo-HSCT) and anti-CD19 chimeric antigen receptor (CAR) T Cell therapy

Cao et al. focus their work on an uncommon complication from allogeneic hematopoietic stem cell transplantation (allo-HSCT) and anti-CD19 chimeric antigen receptor (CAR) T Cell therapy for B Cell lymphoblastic lymphoma/aLL. The authors discussed the development of a cardiac mass and myocardial infiltration, which are associated with increased cytokine levels following allo-HSCT and CAR T Cell therapy. They indicated that monitoring of cardiac functions in B Cell lymphoblastic lymphoma/aLL patients undergoing allo-HSCT and CAR T Cell therapy is of paramount importance.

**FIGURE 1:**
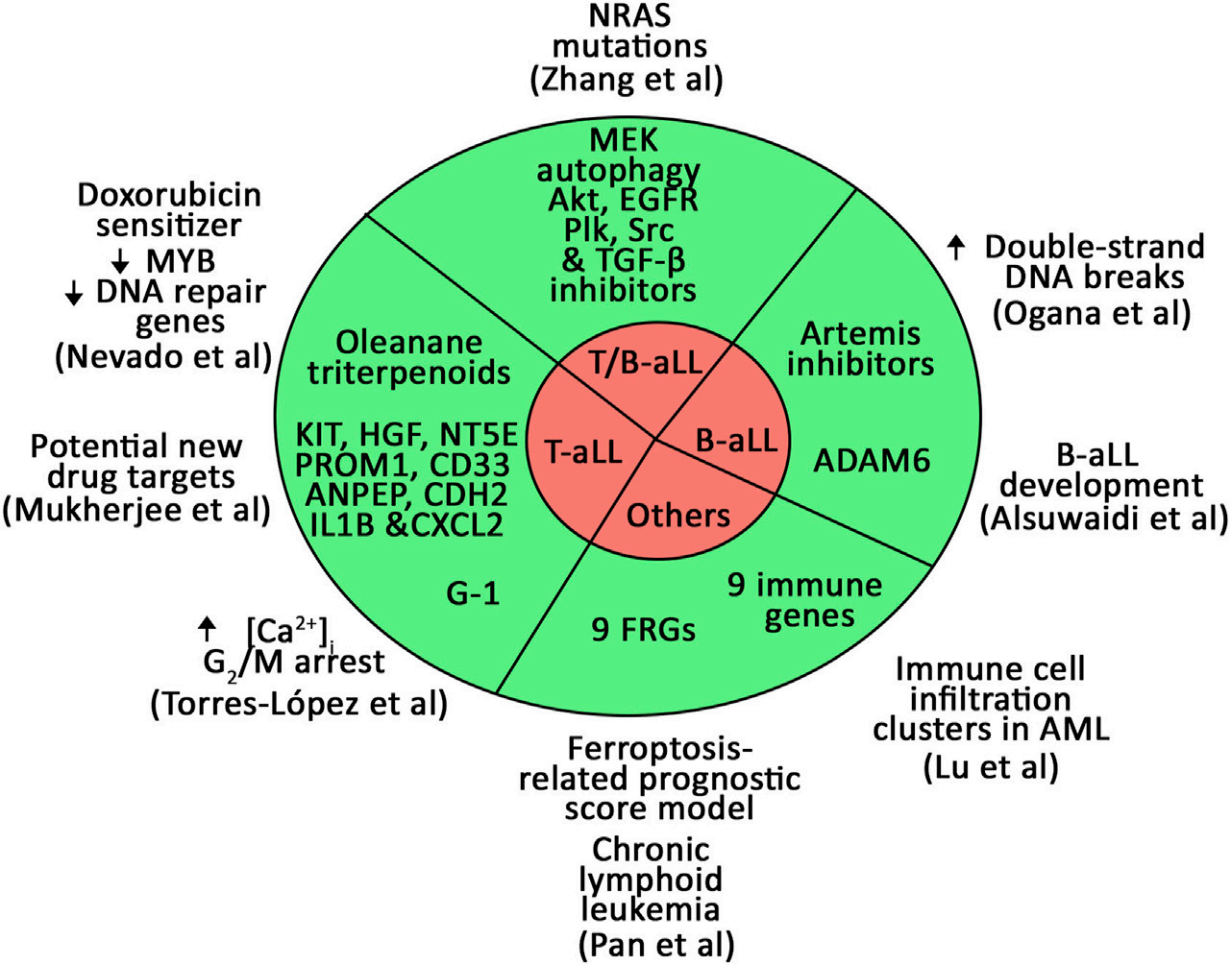
Novel biomarkers and prognostic models for B- and T-aLL and other types of leukemias identified in the current Research Topic.

The current Research Topic highlights new biomarkers and prognostic models for aLL and other types of leukemias (Figure 1). They have the advantage of expanding the potential targets for personalized medicine, which, as it is emerging from clinical practice, paves the way for improved therapy outcome, reducing off-target side effects, and preventing relapse (Rosen et al., 2022).
